# Differences between private and public primary health care centers and differences between men and women in antihypertensive care and cardiovascular prevention in all patients with hypertension treated in primary care in Stockholm County, Sweden

**DOI:** 10.1186/s12875-025-02716-1

**Published:** 2025-01-25

**Authors:** Per Wändell, Anders Norrman, Julia Eriksson, Charlotte Ivarsson, Hrafnhildur Gudjonsdottir, Maria Hagströmer, Lena Lundh, Jan Hasselström, Boel Brynedal, Christina Sandlund, Axel C. Carlsson

**Affiliations:** 1 Division of Family Medicine and Primary Care, Department of Neurobiology, Care Sciences and Society, Karolinska Institutet, Huddinge, Sweden; 2 Academic Primary Health Care Centre, Stockholm Region, Stockholm, Sweden; 3 Division of Biostatistics, Institute of Environmental Medicine, Karolinska Institutet, Stockholm, Sweden; 4 Centre for Epidemiology and Community Medicine, Region Stockholm, Stockholm, Sweden; 5 Department of Global Public Health, Karolinska Institutet, Stockholm, Sweden; 6 Department of Health Promoting Science, Sophiahemmet University, Stockholm, Sweden; 7 Division of Physiotherapy, Department of Neurobiology, Care Sciences and Society, Karolinska Institutet, Huddinge, Sweden

**Keywords:** Antihypertensive agents, Comorbidity, Hypertension, Lifestyle risk reduction, Counseling, Private practice, Sex

## Abstract

**Aims:**

To study differences in cardiovascular prevention and hypertension management in primary care in men and women, with comparisons between public and privately operated primary health care (PHC).

**Methods:**

We used register data from Region Stockholm on collected prescribed medication and registered diagnoses, to identify patients aged 30 years and above with hypertension. Age-adjusted logistic regression was used to calculate odds ratios (ORs) with 99% confidence intervals (99% CIs) using public PHC centers as referents.

**Results:**

In total, 119,267 patients with a registered hypertension diagnosis at their primary care center were included; 58,239 men and 61,028 women. In terms of co-morbidities and medications, there were some differences between privately and publicly run PHC: registered diagnosis of dementia, which was higher at private PHC, age-adjusted OR 1.80 (1.24–2.69). For lifestyle counseling, privately run PHC had a higher rate of registered counseling for tobacco 1.17 (1.06–1.29), physical activity 1.13 (1.06–1.17), unhealthy diet 1.08 (1.04–1.13), and counseling according to highest prioritized level of advice stated by national guidelines 1.14 (1.09–1.18). Differences in comorbidities between men and women were found, with higher frequencies of coronary heart disease, congestive heart failure, atrial fibrillation, stroke, diabetes, and gout among men. Regarding antihypertensive treatment, women received less treatment of calcium channel blockers and ACE inhibitors, but more of angiotensin receptor blockers.

**Conclusions:**

These findings highlight the need for targeted preventive efforts in PHC, especially for male patients, to address disparities in cardiovascular health outcomes. Small differences in preventive measures between public and privately run PHC suggest generally consistent care across healthcare ownership models.

**Supplementary Information:**

The online version contains supplementary material available at 10.1186/s12875-025-02716-1.

## Introduction

Hypertension is a common condition globally as well as the leading risk factor for mortality [1], and is also one of the four major risk factors for coronary heart disease (CHD) [2, 3]. Globally, there has been a shift in blood pressure levels and hypertension during the past decades, from Western high-income countries to low-income South Asian and sub-Saharan African countries, while the blood pressure levels still remain high in central and eastern European countries [4]. However, during the same time period the awareness, treatment, and control of hypertension in high-income countries globally have improved substantially, but with great variation between countries [5]. The prevalence of hypertension has been estimated to be 27% in Sweden but a large proportion of those are undetected [6]. We have previously estimated the prevalence of a hypertension diagnosis in all care forms in a paper based on data from 2011 and five years prior [7]. and the 5-year period prevalence in the total population was 12.2% out of whom 58% had a registered diagnosis in the last studied year, 2011. Identifying hypertension early in order to initiate proper treatment is important to reduce the risk of cardiovascular disease and death.

Most patients with hypertension in Sweden are treated in primary care [7], and hypertension is the second most common registered diagnosis in primary care in Stockholm County [8]. The proportion of patients achieving target blood pressure is low [9], but has increased over time [10]. The focus today is targeting the overall cardiovascular risk profile in each individual, the intention is to reach specific blood pressure targets, changes in lifestyle habits and cardio preventive medical treatment with lipid lowering drugs, anticoagulant and antiplatelet drugs in accordance with national guidelines [11]. Most data on hypertensive care are limited to relatively small study samples or some primary health care (PHC) centers [12], but studies conducted in larger populations are limited. An observed mean blood pressure of 142/80 mmHg was reported in a joint study between a group of PHC in Stockholm County and Västra Götaland County that included almost 75,000 patients with hypertension called Swedish Primary Care Cardiovascular Database (SPCCD) [13]. Yet, previous studies have not solely included patients listed on a PHC and with their hypertension diagnosis registered there.

The primary health care reform in Sweden, giving equal opportunities to publicly and privately run units to provide primary care for citizens has put focus on, whether mode of operation and profit-driven care affects the quality of care in Sweden [14]. There are no data from Sweden available and most international studies have been conducted in hospital settings. A review concluded that “The ‘true’ effect of ownership appears to depend on institutional context, including differences across regions, markets, and over time”, but also that caregivers working for profits often have lower quality, than private non-profit caregivers in the US [15]. To our knowledge, studies on different ownerships of primary care and their effect on the quality of cardio preventive and hypertensive care are lacking in Sweden as well as internationally.

We aimed to study differences in cardiovascular prevention and hypertension management in men and women in primary care, with comparisons between public and privately operated PHC.

## Methods

This was a longitudinal register-based study of all individuals with a registered diagnosis of hypertension in PHC in Stockholm County, Sweden. Stockholm County has 2.4 million residents, which makes up about 20% of the total population of Sweden. In Sweden, most medical care is funded by public health insurance covering all legal residents. Services are provided by the county council, named Region, either at public facilities or by private providers under a contractual agreement with the region. Most residents are registered with a specific PHC center, which enabled us to compare individual level data at different PHC centers. Whether the PHC centers are privately run or publicly run is coded in the register, enabling comparisons of the provided care.

### The Stockholm regional healthcare data warehouse (VAL database)

All residents of Stockholm County are registered in VAL. Both public and private PHC are obligated to record information on all diagnosis codes, and lifestyle counseling codes. A part of the PHC reimbursements is based on registered diagnoses. Data on collected prescriptions in pharmacies are obtained from national registers. At Region Stockholm, this information is automatically collated in the VAL-database, and used for healthcare planning, quality assessment and practice remuneration. Initially, we included all residents in Stockholm County above 30 years of age with at least one hypertension diagnosis (ICD-10 diagnostic codes I10, I11, I12 or I13) registered between 28 of February 2015 and February 29, 2020 at the PHC center where they were registered.

To obtain a high internal validity and only include individuals with hypertensive care in either public or privately run PHC we employed the following additional exclusion criteria: hypertension diagnosis was registered at another health care unit than the one they were registered with (15% were excluded), the individual was not registered with a PHC the last year (9% were excluded), or the individual had not visited their PHC center during the past 18 months (13% were excluded). Patients receiving home care services were not included unless they had a registered contact with their PHC center. We also summarized counseling on lifestyle. For health care related information, please see Supplementary Table 1. All extracted data from VAL was pseudonymized.

### Studied diagnoses, pharmacotherapy, and lifestyle counseling

Hypertension and cardiometabolic diseases were defined as having a registered diagnosis in VAL from primary care in the past 5 years. For detailed information on the definitions of the studied diagnoses, please see Supplementary Table (1) Pharmacotherapy was defined as having collected prescriptions at the pharmacy for the same medicine twice or more in the past 18 months. For detailed information about pharmacotherapy definitions, please see Supplementary Table (2) For lifestyle counseling, we included any counseling (brief counseling, counseling, or advanced counseling) on tobacco, alcohol, physical activity, and unhealthy diet. In order to promote a healthy lifestyle, these three levels of counselling are recommended by the Swedish National Board of Health and Welfare: brief advice, counseling, and advanced counseling. The two levels of counseling talk are based on person centered care. According to the Swedish National Board of Health and Welfare, health care professionals should offer advanced counseling to patients with hypertension who are daily smokers, or patients with unhealthy dietary habits. For those who have a risk use of alcohol, or insufficient physical activity, health care professionals should offer counseling.

### Statistical analyses

Data from VAL was reported as frequencies and differences between men and women were determined by Chi-squared tests. Student´s t-test was used to test the difference in mean age between women and men. With regards to differences in percentages between privately and publicly run PHC, we decided not to make any descriptive statistical tests, as we also provided age-adjusted logistic regression models. Age-adjusted logistic regression models with 99% confidence intervals were used to estimate differences in hypertensive care between private run and publicly run PHC in women and men, with public PHC as referents. Statistical significance was defined as a p-value lower than 0.01, to avoid mass-significance problems as many tests were undertaken. All analyses and data management were conducted using R Statistical Software v. 4.2.0; R Core Team 2022.

## Results

Overall, 119,267 patients with registered hypertension at the PHC center where they were listed and had a registered visit within 3 years February 29, 2020 were included, in total 58,239 men and 61,028 women. Descriptive data are shown in Table 1. Notably, 25% had diabetes, 11% CHD, 12% atrial fibrillation (AF). Most patients were on antihypertensive medication, with 60% on three or more drugs. Calcium channel blockers were most prescribed (44%), thereafter angiotensin receptor blockers (ARB) (43%), beta blockers (41%), ACE inhibitors (36%) and thiazides (22%). Regarding other treatments, statins were prescribed to 59% in patients with concomitant CHD/AF/diabetes/stroke and to 25% in patients without any of these co-morbidities. Acetylsalicylic acid (ASA) was prescribed to 69% in patients with CHD and ASA or anticoagulants in 84% of patients with stroke. Counseling for different lifestyle factors were reported in 3% for tobacco use, 1% for alcohol, 28% for physical activity, 21% for unhealthy diet and holistic or general lifestyle counseling was reported in 28%.

**Table 1:** Comorbidities in the past five years and antihypertensive care in the past 18 months in the total population above 30 years of age with a hypertension diagnosis in the past five years in the Stockholm Region, who have a primary care contact in the last 3 year. Results by sex and for the total population

	Men (*N* = 58239)	Women (*N* = 61028)	*P*-value	Overall (*N* = 119267)
**Age**				
Mean (SD)	67.0 (12.5)	69.9 (12.7)	< 0.001	68.4 (12.7)
Median [Min, Max]	68.0 [30.0, 103]	71.0 [30.0, 109]		70.0 [30.0, 109]
**Diabetes**	16,843 (28.9%)	12,453 (20.4%)	< 0.001	29,296 (24.6%)
**Stroke**	2188 (3.8%)	1752 (2.9%)	< 0.001	3940 (3.3%)
**Coronary heart disease (CHD)**	8702 (14.9%)	4911 (8.1%)	< 0.001	13,613 (11.4%)
**Atrial fibrillation (AF)**	8077 (13.9%)	6238 (10.2%)	< 0.001	14,315 (12.0%)
**Gout**	3863 (6.6%)	1534 (2.5%)	< 0.001	5397 (4.5%)
**Congestive heart failure**	4695 (8.1%)	4049 (6.6%)	< 0.001	8744 (7.3%)
**Dementia**	153 (0.3%)	143 (0.2%)	0.35	296 (0.3%)
**Obesity**	5064 (8.7%)	5792 (9.5%)	< 0.001	10,856 (9.1%)
**Antihypertensive therapy**	51,146 (96.9%)	53,476 (96.4%)	< 0.001	104,622 (96.6%)
**One antihypertensive drug**	1652 (3.1%)	2013 (3.63%)	< 0.001	3665 (3.4%)
**Two antihypertensive drugs**	17,781 (33.7%)	21,403 (38.6%)	< 0.001	39,184 (36.2%)
**Three or more antihypertensive drugs**	33,365 (63.2%)	32,073 (57.8%)	< 0.001	65,438 (60.4%)
**Thiazides**	11,510 (21.8%)	11,852 (21.4%)	0.079	23,362 (21.6%)
**Calcium antagonists**	24,982 (47.3%)	22,937 (41.3%)	< 0.001	47,919 (44.3%)
**Angiotensin receptor blockers**	21,998 (41.7%)	24,858 (44.8%)	< 0.001	46,856 (43.3%)
**ACE inhibitors**	21,622 (41.0%)	17,002 (30.6%)	< 0.001	38,624 (35.7%)
**Beta blockers**	21,321 (40.4%)	22,690 (40.9%)	0.09	44,011 (40.6%)
**Aldosterone antagonists**	2461 (4.7%)	2235 (4.0%)	< 0.001	4696 (4.3%)
**Other potassium saving agents**	26 (0.0%)	38 (0.1%)	0.24	64 (0.1%)
**Aspirin in patients with CHD**	6270 (72.1%)	3181 (64.8%)	< 0.001	9451 (69.4%)
**Statin in patients with CHD/AF/diabetes/stroke**	17,062 (62.8%)	11,189 (54.4%)	< 0.001	28,251 (59.1%)
**Statin in patients without CHD/AF/diabetes/stroke**	7978 (25.7%)	9683 (23.9%)	< 0.001	17,661 (24.7%)
**Anticoagulants or aspirin in patients with stroke**	1858 (84.9%)	1468 (83.8%)	0.36	3326 (84.4%)
**Tobacco counseling**	1810 (3.2%)	2180 (3.7%)	< 0.001	3990 (3.5%)
**Alcohol counseling**	1725 (3.1%)	707 (1.2%)	< 0.001	2432 (2.1%)
**Physical activity counseling**	16,313 (29.1%)	15,678 (26.5%)	< 0.001	31,991 (27.7%)
**Unhealthy diet counseling**	13,284 (23.7%)	11,261 (19.0%)	< 0.001	24,545 (21.3%)
**Counseling lifestyle**	16,839 (30.0%)	15,437 (26.1%)	< 0.001	32,276 (28.0%)

CHD coronary heart disease; AF atrial fibrillation

Regarding sex differences in general (Table 1), women were older, showed lower rate of diabetes, stroke, CHD, AF, and gout, but a slightly higher frequency of registered obesity. Regarding medications, more women than men were treated by two antihypertensive drugs, and fewer were on three antihypertensive drugs. For specific antihypertensives, women received less calcium receptor blockers, ACE inhibitors, but more ARBs, while treatment with thiazides, beta blockers and aldosterone antagonists were fairly equal. Regarding other medications, women received less treatment with statins, and less treatment with ASA in patients with CHD, slightly less anticoagulant treatment in patients with AF, but fairly equal treatment with ASA or anticoagulants in patients with stroke. All types of lifestyle counselling codes were more common in men than women. There were similar findings for men and women in private and public run PHCCs.

We observe small differences in the prevalence of comorbidities and medication usage between patients managed in private and public run PHC. A marginally higher frequency of registered diagnosis of dementia was found in private PHC a somewhat higher frequency of statin treatment was seen in privately run PHC, when compared to publicly run PHC (Table 2).

**Table 2:** Characteristics of men and women in primary care in Stockholm Region by 2020-02-29 divided into private care and public driven care

	Men		Women		Total	
	Public (*N* = 16911)	Private (*N* = 41109)	Public (*N* = 17701)	Private (*N* = 43089)	Public (*N* = 34612)	Private (*N* = 84198)
Age						
**Mean (SD)**	67.0 (12.5)	67.0 (12.5)	69.9 (12.6)	69.9 (12.7)	68.4 (12.6)	68.5 (12.7)
**Median [Min**,** Max]**	68.0 [31.0, 100]	68.0 [30.0, 103]	71.0 [31.0, 107]	71.0 [30.0, 109]	70.0 [31.0, 107]	70.0 [30.0, 109]
**Diabetes**	4859 (28.7%)	11,927 (29.0%)	3483 (19.7%)	8929 (20.7%)	8342 (24.1%)	20,856 (24.8%)
**Stroke**	639 (3.8%)	1539 (3.7%)	507 (2.9%)	1240 (2.9%)	1146 (3.3%)	2779 (3.30%)
**Coronary heart disease CHD**	2526 (14.9%)	6146 (15.0%)	1455 (8.2%)	3440 (8.0%)	3981 (11.5%)	9586 (11.4%)
**Atrial fibrillation** (AF)	2427 (14.4%)	5631 (13.7%)	1771 (10.0%)	4454 (10.3%)	4198 (12.1%)	10,085 (12.0%)
**Gout**	1155 (6.8%)	2696 (6.6%)	449 (2.5%)	1076 (2.5%)	1604 (4.6%)	3772 (4.50%)
**Congestive heart failure**	1406 (8.3%)	3278 (8.0%)	1151 (6.5%)	2891 (6.7%)	2557 (7.4%)	6169 (7.3%)
**Dementia**	35 (0.2%)	117 (0.3%)	20 (0.1%)	123 (0.3%)	55 (0.2%)	240 (0.3%)
**Obesity**	1500 (8.9%)	3527 (8.6%)	1656 (9.4%)	4097 (9.5%)	3156 (9.1%)	7624 (9.1%)
**Antihypertensive therapy**	14,767 (96.8%)	36,179 (96.9%)	15,434 (96.3%)	37,840 (96.4%)	30,201 (96.6%)	74,019 (96.6%)
**One antihypertensive drug**	482 (3.2%)	1165 (3.1%)	593 (3.7%)	1412 (3.6%)	1075 (3.4%)	2577 (3.4%)
**Two antihypertensive drug**s	5039 (33.0%)	12,668 (33.9%)	6171 (38.5%)	15,140 (38.6%)	11,210 (35.8%)	27,808 (36.3%)
**Three** or **more antihypertensive drug**s	9728 (63.8%)	23,511 (63.0%)	9263 (57.8%)	22,700 (57.8%)	18,991 (60.7%)	46,211 (60.3%)
**Thiazides**	3295 (21.6%)	8175 (21.9%)	3309 (20.6%)	8509 (21.7%)	6604 (21.1%)	16,684 (21.8%)
**Calcium antagonists**	7397 (48.5%)	17,498 (46.9%)	6713 (41.9%)	16,144 (41.1%)	14,110 (45.1%)	33,642 (43.9%)
**Angiotensin receptor blockers**	6344 (41.6%)	15,573 (41.7%)	7142 (44.6%)	17,605 (44.9%)	13,486 (43.1%)	33,178 (43.3%)
**A**CE **inhibitors**	6222 (40.8%)	15,301 (41.0%)	4914 (30.7%)	12,027 (30.6%)	11,136 (35.6%)	27,328 (35.7%)
**Beta blockers**	6174 (40.5%)	15,066 (40.3%)	6523 (40.7%)	16,098 (41.0%)	12,697 (40.6%)	31,164 (40.7%)
**Aldosterone antagonists**	761 (5.0%)	1692 (4.5%)	662 (4.1%)	1565 (4.0%)	1423 (4.6%)	3257 (4.3%)
**Other potassium saving agents**	10 (0.1%)	16 (0.0%)	13 (0.1%)	25 (0.1%)	23 (0.1%)	41 (0.1%)
**Aspirin in patients with CHD**	1807 (71.5%)	4440 (72.2%)	936 (64.3%)	2234 (64.9%)	2743 (68.9%)	6674 (69.6%)
**Statin in patients with CHD**/**AF**/**diabetes**/**stroke**	4863 (61.4%)	12,148 (63.4%)	3099 (53.1%)	8060 (54.9%)	7962 (57.9%)	20,208 (59.7%)
**Statin in patients without CHD**/**AF**/**diabetes**/**stroke**	2164 (24.1%)	5783 (26.4%)	2579 (21.7%)	7074 (24.9%)	4743 (22.7%)	12,857 (25.5%)
**Anticoagulants** or **asp**i**rin in patients with stroke**	551 (86.2%)	1300 (84.5%)	426 (84.0%)	1038 (83.7%)	977 (85.3%)	2338 (84.1%)
**Tobacco counseling**	451 (2.8%)	1355 (3.4%)	586 (3.4%)	1577 (3.8%)	1037 (3.1%)	2932 (3.6%)
**Alcohol counseling**	484 (3.0%)	1236 (3.1%)	196 (1.14%)	509 (1.2%)	680 (2.0%)	1745 (2.1%)
**Physical activity counseling**	4500 (27.7%)	11,751 (29.7%)	4210 (24.5%)	11,409 (27.3%)	8710 (26.0%)	23,160 (28.4%)
**Unhealthy eating counseling**	3716 (22.9%)	9516 (24.0%)	3093 (18.0%)	8137 (19.5%)	6809 (20.4%)	17,653 (21.7%)
**Counseling lifestyle**	4623 (28.4%)	12,156 (30.7%)	4141 (24.1%)	11,247 (26.9%)	8764 (26.2%)	23,403 (28.7%)

CHD coronary heart disease; AF atrial fibrillation

Age- and comorbidity adjusted odds ratios for the differences between public and privately run PHC are shown in Table 3. All types of registered lifestyle counseling were observed to a slightly higher frequencies in privately run PHC. The differences between public and privately run PHC centers did not differ between the sexes.

**Table 3:** The effect of mode of operation, private vs. publicly (referents) run primary health care center on registered antihypertensive care and outcomes

	Women				Men				Women and men		
	OR	99% CI	*P*-values^*^		OR	99% CI	*P*-values^*^		OR	99% CI	*P*-values^*^
**Diabetes**	**1.07**	**1.01**,** 1.13**	**0.004**		1.01	0.96, 1.07	0.5		1.04	1.00, 1.08	0.016
**Stroke**	1.00	0.87, 1.15	0.9		0.99	0.87–1.12	0.8		0.99	0.91, 1.09	0.9
**Coronary heart disease (CHD)**	0.96	0.89, 1.05	0.3		1.00	0.93, 1.07	0.9		0.99	0.94, 1.04	0.5
**Atrial fibrillation (AF)**	1.04	0.96, 1.12	0.3		**0.94**	**0.88**,** 1.01**	**0.023**		0.98	0.93, 1.03	0.3
**Gout**	0.98	0.85, 1.14	0.7		0.96	0.87, 1.05	0.2		9.96	0.89, 1.04	0.2
**Chronic heart failure**	1.03	0.94, 1.14	0.4		0.95	0.87, 1.04	0.14		0.99	0.93, 1.05	0.6
**Dementia**	**2.53**	**1.42**,** 4.97**	**< 0.001**		1.38	0.85, 2.32	0.10		**1.80**	**1.24**,** 2.69**	**< 0.001**
**Obesity**	1.02	0.94, 1.11	0.5		0.97	0.89, 1.05	0.3		0.99	0.94, 1.05	0.8
**Antihypertensive therapy**	1.03	0.90, 1.17	0.6		1.01	0.88, 1.17	0.8		1.02	0.93, 1.12	0.6
**One antihypertensive drug**	0.97	0.85, 1.11	0.6		0.99	0.86, 1.14	0.8		0.98	0.89, 1.08	0.6
**Two antihypertensive drugs**	1.00	0.95, 10.5	0.9		1.04	0.99, 1.10	0.055		1.02	0.98, 1.06	0.2
**Three or more antihypertensive drugs**	1.00	0.95, 10.5	0.9		0.96	0.92, 1.02	0.074		0.98	0.95, 1.02	0.2
**Thiazides**	1.06	1.00, 1.13	0.007		1.02	0.96, 1.08	0.5		1.04	1.00, 1.09	0.016
**Calcium antagonists**	0.97	0.92, 1.02	0.10		0.94	0.89, 0.98	< 0.001		0.95	0.92, 0.99	< 0.001
**Angiotensin receptor blockers**	1.01	0.86, 1.06	0.5		1.00	0.95, 1.06	0.8		1.01	0.97, 1.04	0.6
**ACE inhibitors**	1.00	0.95, 1.05	0.9		1.01	0.96, 1.06	0.7		1.00	0.97, 1.04	0.8
**Beta blockers**	1.01	0.96, 1.07	0.5		0.99	0.94, 1.05	0.8		1.00	0.97, 1.04	0.8
**Aldosterone antagonists**	0.96	0.85, 1.09	0.4		0.90	0.81, 1.02	0.024		0.93	0.86, 1.01	0.030
**Other potassium saving agents**	0.78	0.33, 2.00	0.5		0.65	0.24, 1.97	0.3		0.73	0.38, 1.46	0.2
**Aspirin in patients with CHD**	1.04	0.88, 1.23	0.6		1.04	0.90, 1.19	0.5		1.04	0.93, 1.16	0.3
**Statin in patients with CHD/AF/diabetes/stroke**	1.07	0.99, 1.16	0.021		1.09	1.01, 1.17	0.002		1.08	1.02, 1.14	< 0.001
**Statin in patients without CHD/AF/diabetes/stroke**	1.20	1.12, 1.28	< 0.001		1.13	1.04, 1.22	< 0.001		1.17	1.11, 1.23	< 0.001
**Anticoagulants or aspirin in patients with stroke**	0.98	0.67, 1.41	0.9		0.87	0.61, 1.22	0.3		0.92	0.71, 1.18	0.4
**Tobacco counseling**	1.11	0.98, 1.27	0.030		1.24	1.08, 1.44	< 0.001		1.17	1.06, 1.29	< 0.001
**Alcohol counseling**	1.07	0.86, 1.33	0.4		1.05	0.91, 1.21	0.4		1.06	0.94, 1.19	0.2
**Physical activity counseling**	1.16	1.10, 1.22	< 0.001		1.10	1.05, 1.16	< 0.001		1.13	1.09, 1.17	< 0.001
**Unhealthy eating counseling**	1.10	1.04, 1.17	< 0.001		1.07	1.01, 1.13	0.003		1.08	1.04, 1.13	< 0.001
**Counseling lifestyle**	1.16	1.10, 1.22	< 0.001		1.11	1.06, 1.18	< 0.001		1.14	1.09, 1.18	< 0.001

CHD coronary heart disease; AF atrial fibrillation

^*^ Based on a age-adjusted logistic regression model

## Discussion

We included patients with hypertension and only individuals where the hypertension was registered and cared for in PHC. The pattern of co-morbidities in men and women with hypertension was in line with what has been reported in the literature where patients with hypertension has been extracted and their care have been assessed without ensuring registration in PHC. There were expected differences between men and women when it comes to cardiovascular comorbidities and diabetes being higher in men and that some antihypertensive drugs were more common in women. About 60% of the patients were treated with three or more antihypertensive drugs. There were little differences between public and privately run PHC, where the main difference was found to be that more registered lifestyle counseling was found in privately run PHC.

Differences in co-morbidities between men and women have been described earlier and are well known [16]. Women seek care more often than men [8], while men have shorter life expectancy, and get their cardiac diseases around 5 years earlier than women [17, 18], and diabetes 2–4 years earlier [19]. We found more women than men with hypertension treated in PHC which may be a result of a higher care seeking behavior and that they were detected opportunistically. The phenomenon that health seeking behavior and the disease burden have opposite directions in men and women, respectively, have been described as a health paradox [20]. An international meta-analysis has shown that a larger proportion of men (51%) compared to women (41%) with hypertension were unaware of their diagnosis [21]. This indicates that screening might occur to a smaller extent among men, and that hypertension is detected later leading to more complications. The higher frequencies of diabetes, gout, stroke, CHD, AF, and CHF in men are thus to be expected [22]. As regards obesity, this diagnosis is seldom set, and an under-reporting is most probably present. Dementia diagnoses were fewer than expected. This may be due to under reporting as well as patients not receiving the diagnosis while being cared for in the home care system unless also visiting the PHC. A novelty with the present study verses previous studies comparing care of men and women was that we only included individuals with a hypertension diagnosis in the PHC where they were listed, as well as receiving their care in the past year. In previous studies in Swedish PHC, the care has been assessed without ensuring that the hypertension is known and registered recently [13].

Although there are no specific recommendations when it comes to antihypertensive treatment in any sex, we found many differences that have been described previously: that women were more often treated with diuretics, and men with calcium channel blockers and ACE-inhibitors [23, 24]. In the present study, we found higher rates among men using calcium channel blockers and ACE inhibitors, but a lower rate of ARBs; and higher rates among men with hypertension and concomitant CHD using ASA (aspirin).

Previous studies comparing hypertension care between private and public PHC units, mostly from other parts of the world with other health care systems, have shown better results in privately run PHC units [25, 26]. In contrast, we found only marginal differences between private and public run PHC, mostly regarding registered counseling for lifestyle and risk factors which was more commonly registered in privately run PHC. Lifestyle counselling has been associated with better cardio-preventive health. The rate of registered counseling for lifestyle and risk factors is low. This is likely due to a low rate of registrations, and the result is not reflecting that the conversations are not taking place, as personal communication with health care professionals is often not reported when another reason such as a diagnosis is reported for the visit.

We have previously studied lifestyle counseling [27, 28]. One possible explanation for the higher rates of recorded counseling for lifestyle and risk factors among privately run PHC could be a consequence of privately run PHC being keener to register these, owing to the reimbursement system but incitements to report diagnoses and efforts are high in publicly run PHC as well. A higher rate of statin use was found in privately run PHC, both for use in indicated medical disorders where statin use is recommended, but also outside these diagnoses, where statin therapy are not as widely recommended as in the groups with a high cardiovascular risk [29]. We know of no previous study examining the prescription of drugs in private and publicly founded PHC.

There was a higher rate of patients with dementia at the privately run PHC is surprising even if the overall rates were low and underreported. Patients at nursing homes are mostly listed at their nursing homes. We can’t exclude that some nursing homes are provided with doctors from the private owned PHC and could be listed at these PHC centers. It is estimated that there are130-150,000 individuals with dementia in Sweden [30], and the estimated prevalence of dementia in Region Stockholm should be around 2%.

There are some limitations with this study. We did not have access to registered blood pressure values and the extent to which patients achieve target blood pressure. Patients included were listed at specific PHC center and had registered visits. We did not have access to patients who are not listed but that is less than 10% of the population in Stockholm. Patients at nursing homes should have access to medical care from PHC, but were excluded from the present study, as they are not listed as in care at the PHC but at their nursing home. Additionally, patients were excluded if they did not have their hypertension diagnosis registered at their PHC where they were registered (15%), not being registered (9%) or not having visited their PHC center during the last year (13%). Regarding lifestyle counseling, only registered counseling codes was included, and the total number of counseling, above all brief advice, may probably be underreported in all PHC. Lifestyle counselling might be recorded in the free text in the electronic patient records, but this information was not accessible in the present study.

One of the strengths with this study was that we had access to all patients attending PHC regularly, which is important as most patients with hypertension receive their care in PHC [31]. To assure fair comparisons between private and public care, we excluded patients with a hypertension diagnosis registered in another PHC than they were registered at, those not registered with a PHC and those whom had not visited their PHC in the past 18 months.

We conclude, that within this comparison between private and publicly run PHC, there were only limited differences, mostly in registered lifestyle counseling; suggesting that hypertensive care is of similar quality in privately run versus publicly run PHC. Differences between men and women are in line with earlier findings, with higher rates of cardio-metabolic diseases among men, including CHD, CHF, AF, stroke, diabetes, and gout. There is room for improvements in the care of patients with hypertension. Studies including blood pressure data on all individuals listed on public and private PHC are warranted to determine if there are differences in control as well as in opportunistic detection of hypertension.

## Electronic supplementary material

Below is the link to the electronic supplementary material.

Supplementary Material 1

## Data Availability

The data from this study can be accessed for research by qualified researchers who have been trained in confidentiality protocols for human subjects, following ethical approval from Region Stockholm at halsodata.rst@regionstockholm.se.
