# Supplementary material for: Differences between private and public primary health care centers and differences between men and women in antihypertensive care and cardiovascular prevention in all patients with hypertension treated in primary care in Stockholm County, Sweden
Source: BMC Prim Care. 2025 Jan 25;26:20. doi: 10.1186/s12875-025-02716-1 (PMC11763146; doi:10.1186/s12875-025-02716-1)
Supplement: Supplementary file 1 — Supplementary Material 1 [file 12875_2025_2716_MOESM1_ESM.docx]

# Supplementary Table 1 Diagnosis codes, Measurements of care

## Hypertension

- ICD I10 Essential (primary) hypertension
- ICD I11 Hypertensive heart disease
- ICD I12 Hypertensive renal disease
- ICD I13 Hypertensive heart and renal disease

## Comorbidity diagnosis

- Ischaemic heart diseases (I20-I25)

I20.0, I20.1, I20.8, I20.9

I21.0, I21.1 I21.2, I21.3, I21.4, I21.9

I22.0, I22.1, I22.8, I22.9

I23.0, I23.1, I23.2, I23.4, I23.5, I23.6, I23.8

I24.0, I24.1, I24.8, I24.9

I25.0, I25.1, I25.2, I25.3, I25.4, I25.5, I25.6, I25.8, I25.9

- Diabetes (E10-E14)

E10.0, E10.1, E10.2, E10.3, E10.4, E10.5, E10.6, E10.7, E10.8, E10.9

E11.0, E11.1, E11.2, E11.3, E11.4, E11.5, E11.6, E11.7, E11.8, E11.9

E12.0, E12.1, E12.2, E12.3, E12.4, E12.5, E12.6, E12.7, E12.8, E12.9

E13.0, E13.1, E13.2, E13.3, E13.4, E13.5, E13.6, E13.7, E13.8, E13.9

E14.0, E14.1, E14.2, E14.3, E14.4, E14.5, E14.6, E14.7, E14.8, E14.9

- Atrial fibrillation and flutter (I48)

I48.0, I48.1, I48.2, I48.3, I48.4, I48.9

- Heart failure (I50)

I50.0, I50.1, I50.9

- Cerebrovascular diseases (I61-I64, I69)

I61.0, I61.1, I61.2, I61.3, I61.4, I61.5, I61.6, I61.8, I61.9

I62.0, I62.1, I62.9

I63.0, I63.1, I63.2, I63.3, I63.4, I63.5, I63.6, I63.8, I63.9

I64.9

I69.0, I69.1, I69.2, I69.3, I69.4, I69.8

- Depression (F32-F33)

F32.0, F32.1, F32.2, F32.3, F32.4, F32.8, F32.9

F33.0, F33.1, F33.2, F33.4, F33.8, F33.9

- Gout (M10)

M10.0, M10.1, M10.2, M10.3, M10.4, M10.9

- Obesity (E65-E68)

E65.9

E66.0, E66.1, E66.2, E66.8, E66.9

## Lifestyle diagnosis

- Tobacco

Z72.0 Tobacco use

F17.1 Mental and behavioural disorders due to use of tobacco

F17.2 Mental and behavioural disorders due to use of tobacco : dependence syndrome

- Risky use of alcohol

Z72.1 Alcohol use

F10.1 Mental and behavioural disorders due to use of alcohol : harmful use

F10.2 Mental and behavioural disorders due to use of alcohol : dependence syndrome

- Insufficient physical activity

Z72.3 Lack of physical exercise

- Unhealthy eating habits

Z72.4 Inappropriate diet and eating habits

## Lifestyle advice

- Tobacco

DV111 Enkla råd

DV112 Rådgivande samtal

**DV113 Kvalificerat rådgivande samtal**

- Risky use of alcohol

DV121 Enkla råd

**DV122 Rådgivande samtal**

DV123 Kvalificerat rådgivande samtal

- Insufficient physical activity

DV131 Enkla råd

**DV132 Rådgivande samtal**

DV133 Kvalificerat rådgivande samtal

DV200 Utfärdande av Fysisk aktivitet på recept

AW005 Uppföljning av tidigare utfärdat recept på fysisk aktivitet (FaR)

- Unhealthy eating habits

DV141 Enkla råd

DV142 Rådgivande samtal

**DV143 Kvalificerat rådgivande samtal**

# Supplementary table 2 Collected prescribed pharmacotherapy

| **C02 Antihypertensive drugs** | C02AC01 Klonidin  C02AC02 Guanfacin  C02AC05 Moxinidin  C02CA04 Doxazosin  C02DB02 Hydralazin  C02KX01 Bosentan (PAH-lm)  C02KX02 Ambrisentan (PAH-lm)  C02KX04 Macitentan (PAH-lm)  C02KX05 Riociguat |
| --- | --- |
| **C03 Diuretics** | C03AA01 Bendroflumetiazid  C03AA03 Hydroklotriazid  C03AB01 Bendroflumetiazid och kalium  C03BA04 Klortalidon?  C03BA08 Metolazon?  C03CA01 Furosemid  C03CA02 Bumetanid  C03CA04 Torasemid?  C03DA01 Spironolakton  C03DA04 Eplerenon  C03EA01 Hydroklortiazid och kaliumsparande lm  C03XA01 Tolvaptan |
| **C07 Beta receptor blockers** | C07AA05 Propranolol  C07AA07 Sotalol  C07AB02 Metoprolol  C07AB03 Atenolol  C07AB07 Bisoprolol  C07AB09 Esmolol  C07AB14 Landiolol  C07AG01 Labetalol  C07AG02 Karvedilol  C07FB02 Metoprolol och felodipin |
| **C08 Calcium antagonists** | C08CA01 Amlodipin  C08CA02 Felodipin  C08CA05 Nifedipin  C08CA06 Nimodipin  C08CA13 Lerkanidipin  C08CA16 Klevidipin  C08DA01 Verapamil  C08DB01 Diltiazem |
| C09 Medications affecting the renin-angiotensin system | C09AA01 Kaptopril  C09AA02 Enalapril  C09AA03 Lisinopril  C09AA04 Perindopril  C09AA05 Ramipril  C09AA09 Fosinopril  C09CA01 Losartan  C09CA02 Eprossartan  C09CA03 Valsartan  C09CA04 Irbesartan  C09CA06 Kandesartan  C09CA07 Telmisartan  C09CA08 Olmesartanmedoxomil  C09CA09 Azilsartanmedoxomil  C09XA02 Aliskiren  C09BA02 Enalapril och diuretika  C09BA03 Lisinopril och diuretika  C09BA05 Ramipril och diuretika  C09BA06 Kinapril och diuretika  C09DA01 Losartan och diuretika  C09DA02 Eprosartan och diuretika  C09DA03 Valsartan och diuretika  C09DA04 Ibersartan och diuretika  C09DA06 Kandesartan och diuretika  C09DA07 Telmisartan och diuretika  C09XA52 Aliskiren och hydroklortiazid  C09DB01 Valsartan och amlodipin  C09DB04 Telmisartan och amlodipin  C09DX01 Valsartan, amlodipin och hydroklortiazid  C09DX04 Valsartan och sakubitril  C09BB02 Enalapril och lerkanidipin  C09BB07 Ramipril och amlodipin |
| **C10 Medications affecting the serum lipid levels** | C10AA01 Simvastatin  C10AA03 Pravastatin  C10AA04Fluvastatin  C10AA05 Atorvastatin  C10AA07 Rosuvastatin  C10AA08 Pitavastatin  C10BA02 Simvastatin och ezetimib  C10BA03 Pravastatin och fenofibrat  C10BA04 Simvastatin och fenofibrat  C10BA05 Atorvastatin och ezetimib  C10BA06 Rosuvastatin och ezetimib  C10BA10 Bempedinsyra och ezetimid |
| **B01 Anticoagulants** | B01AA03 Warfarin  B01AB01 Heparin  B01AB02 Antitrombin III  B01AB04 Dalteparin  B01AB05 Enoxparin  B01AB09 Danaparoid  B01AB10 Tinzaparin  B01AC04 Klopidogrel  B01AC05 Tiklopidin  B01AC06 Acetylsalicylsyra  B01AC07 Dipyridamol  B01AC09 Epoprostenol  B01AC11 Iloprost  B01AC16 Eptifibatid  B01AC17 Tirofiban  B01AC21 Treprostinil  B01AC22 Prasugrel  B01AC23 Cilostazol  B01AC24 Tikagrelol  B01AC25 Kangrelol  B01AC27 Selexipag  B01AC30 Trombocytaggregationshämmande medel, kombinationer  B01AE03 Argatroban?  B01AE06 Bivalirudin?  B01AE07 Dabigatranetexilat  B01AF01 Rivaroxaban  B01AF02 Apixaban  B01AF03 Edoxaban  B01AX01 Defibrotid  B01AX05 Fondaparinux  B01AX07 Kaplacizumab |
| **A10 Diabetes medications** | A10AB01 Insulin  A10AB04 Insulin, lispro  A10AB05 Insulin, aspart  A10AB06 Insulin, glulisin  A10AC01 Insulin  A10AC04 insulin, lispro  A10AD01 Insulin  A10AD04 Insulin, lispro  A10AD05 Insulin, aspart  A10AD06 Insulin, degludek och insulin, aspart  A10AE04 Insulin, glargine  A10AE05 Insulin, detemir  A10AE06 Insulin, degludek  A10AE54 Insulin, grargin och lixisenatid  A10AE56 Insulin, degludek och liraglutid  A10BA02 Metformin  A10BB01 Glibenklamid  A10BB07 Glipizid  A10BB12 Glimepirid  A10BD05 Metformin och pioglitazon  A10BD06 Glimepirid och pioglitazon  A10BD07 Metformin och sitagliptin  A10BD08 Metformin och vildagliptin  A10BD09 Pioglitazon och alogliptin  A10BD10 Metformin och saxagliptin  A10BD11 Metformin och linagliptin  A10BD13 Metformin och alogliptin  A10BD15 Metformin och dapagliflozin  A10BD16 Metformin och kanaligliflozin  A10BD19 Linagliptin och empagliflozin  A10BD20 Metformin och empagliflozin  A10BD21 Saxagliptin och dapagliflozin  A10BD23 Metformin och ertugliflozin  A10BD24 Sitagliptin och ertugliflozin  A10BF01 Akarbos  A10BD03 Pioglitazon  A10BH01 Sitagliptin  A10BH02 Vildagliptin  A10BH03 Saxagliptin  A10BH04 Alogliptin  A10BH05 Linagliptin  A10BJ01 Exenatid  A10BJ02 Liraglutid  A10BJ03 Lixisenatid  A10BJ05 Dulaglutid  A10BJ06 Semaglutid  A10BK01 Dapagliflozin  A10BK02 kanagliflozin  A10BK03 Empagliflozin  A10BK04 Ertugliflozin  A10BK06 Sotagliflozin  A10BX02 Repaglinid  A10BX03 Nateglinid |
| **M04 Gout medications** | M04AA01 Allopurinol  M04AA03 Febuxostat  M04AB01 Probenecid  M04AC01 Kolkicin |
